# Validation of the ECTemp™ algorithm in construction workers: a comparison of photoplethysmography smartwatch and chest strap heart rate monitors

**DOI:** 10.1088/2515-7620/ae7bc5

**Published:** 2026-06-19

**Authors:** Maxime Jeanovitch Lignier, Erica Tourula, Jennifer M Raef, Jonathan W Specht, Alyssa Bailly, M Jo Hite, Charlie Walker, Leo Sweitzer, Hillary A Yoder, Serena Garcia, Omkar Khandpekar, Roger S Zoh, Jason Glaser, David H Wegman, Fabiano Amorim, Zachary J Schlader

**Affiliations:** 1 Department of Kinesiology, Indiana University School of Public Health—Bloomington, Bloomington, IN, United States of America; 2 Department of Physiology and Anatomy, University of North Texas Health Fort Worth, Fort Worth, TX, United States of America; 3 Department of Health, Exercise and Sports Sciences, University of New Mexico, Albuquerque, NM, United States of America; 4 Department of Kinesiology & Athletic Training, University of Northern Iowa, Cedar Falls, IA, United States of America; 5 Department of Kinesiology, New Mexico State University, Las Cruces, NM, United States of America; 6 Department of Epidemiology and Biostatistics, Indiana University School of Public Health—Bloomington, Bloomington, IN, United States of America; 7 La Isla Network, Washington, DC, United States of America; 8 Department of Public Health, University of Massachusetts- Lowell, Lowell, MA, United States of America

**Keywords:** estimated core temperature, wearable devices, photoplethysmography (PPG), ECTemp algorithm, construction workers, occupational heat stress, field validation

## Abstract

*Background.* Core temperature monitoring may provide information to mitigate heat-related illnesses among construction workers. This study evaluated the validity of the ECTemp™ algorithm and compared the use of a photoplethysmography-based smartwatch to a chest strap as the heart rate input for the algorithm. *Methods.* In 102 construction workers during the summer, gastrointestinal temperature was measured via ingestible telemetric pill (*T*_PILL_), and heart rate was measured with a Polar H10 chest strap (*n* = 102) and a Garmin Instinct 2 smartwatch (*n* = 60) during a single work shift. Peak, mean and continuous time-series data from each device were used to compute core temperature estimates using ECTemp™ (ECTemp_GARMIN_ and ECTemp_POLAR_). Agreement was evaluated with Bland–Altman analyses, Pearson correlations, and Kendall’s Tau correlations. *Results.* Compared to T_PILL_, ECTemp_POLAR_ showed a bias of −0.16 ± 0.30 °C for continuous data, with a median Kendall’s τ of 0.39. ECTemp_GARMIN_ showed a bias of −0.09 °± 0.28 °C (median *τ* = 0.36). The two devices produced similar heart rate measures (bias: 1.6 ± 4.9 beats min^−1^; median *τ* = 0.75) and ECTemp outputs (bias: 0.03 °± 0.06 °C; median *τ* = 0.83). *Conclusion.* ECTemp_POLAR_ and ECTemp_GARMIN_ are both adequate estimates of core temperature for construction work monitoring at a group level. The more practical ECTemp_GARMIN_ appeared to have less bias than ECTemp_POLAR_, perhaps warranting future consideration for field use.

## 1. Introduction

Worldwide, billions of people regularly encounter work-related heat stress (WrHS) (Kjellstrom *et al* 2019, Azzi *et al* 2024). WrHS stems from a combination of physical exertion and hot environmental conditions and/or protective clothing, increasing physiological strain via the development of hyperthermia (i.e. increases in core temperature) (Schlader *et al* 2017, Ioannou *et al* 2022, Masoud *et al* 2024), the latter of which can be potentiated by dehydration (Chapman *et al* 2020). WrHS-induced hyperthermia is associated with adverse health effects including an increased risk of injuries, illness and death (Azzi *et al* 2024). Notably, construction workers are disproportionately affected. For example, despite construction workers making up only 6% of the American workforce, they comprise a third of all WrHS-related injuries/illnesses (Hawkins and Ibrahim, 2023) and WrHS-related deaths (Dong *et al* 2019) in the U.S.

Given the disproportionate impact of WrHS-induced hyperthermia on construction workers, reliable core temperature monitoring on worksites may be needed to enable timely rest-shade-hydration interventions (Bodin *et al* 2016, Wegman *et al* 2018, Glaser *et al* 2020, 2022, Hansson *et al* 2024, Masoud *et al* 2024). Previous field studies primarily utilized ingestible temperature-sensitive pills (*T*_PILL_) to measure gastrointestinal temperature (Moyce *et al* 2017, McKenna *et al* 2023). These pills provide a valid surrogate measure of core temperature (Bongers *et al* 2018), which in controlled laboratory or clinical settings can be measured with the gold standard methods of rectal or esophageal probes (Patel *et al* 1996, Lee *et al* 2000, Lefrant *et al* 2003, Casa *et al* 2007). While *T*_PILL_ is more practical, it is not without limitations: the pills are expensive, require specialized equipment, can pass the gastrointestinal tract prematurely (Hertzberg *et al* 2017, Krisher *et al* 2025), and their measurements fluctuate when hot or cold drinks are consumed (Wilkinson *et al* 2008).

A promising non-invasive alternative is the ECTemp™ algorithm, which provides real-time estimated core temperature (ECTemp^TM^) based on continuous measurements of heart rate (Buller *et al* 2013, Looney *et al* 2018). This algorithm has been validated in military settings (Buller *et al* 2013, Hunt *et al* 2019), in first responders (Buller *et al* 2015) and athletes (Hagen *et al* 2020, de Korte *et al* 2022), during prolonged walking exercise (Peggen *et al* 2024), and in agricultural work settings (Egbert *et al* 2022). Based on its validation in other settings, the use of ECTemp™ in construction could be beneficial in monitoring heat strain. To our knowledge, however, no prior studies have investigated the validity of using the ECTemp™ algorithm in construction workers.

Most research attempting to assess the validity of the ECTemp™ algorithm has used chest strap heart rate monitors such as Equivital EQ02™, Hidalgo (Buller *et al* 2013, 2015), Zephyr Bioharness 3™ from Medtronic (Hagen *et al* 2020), HRM-Run™ from Garmin (Peggen *et al* 2024) or various Polar™ models (Hunt *et al* 2019, Egbert *et al* 2022). Chest strap heart rate monitors measure the electrical responses of the heart and are established as accurate (Laukkanen and Virtanen 1998, Achten and Jeukendrup 2003). However, they can be cumbersome and uncomfortable to wear regularly (Beeler *et al* 2018). Smartwatches could offer a more practical alternative, potentially improving daily user adherence in occupational settings like construction. These devices measure pulse rate as a surrogate for heart rate using photoplethysmography (PPG), the noninvasive measurement of blood volume changes in the microvasculature (Allen 2007). Previous studies have shown mixed validity with PPG, with estimates of heart rate from smartwatches generally being inferior to chest straps (Dooley *et al* 2017, Pasadyn *et al* 2019, Støve *et al* 2019, Düking *et al* 2020, Fuller *et al* 2020, Navalta *et al* 2020, 2023, 2024, Cosoli *et al* 2022, Germini *et al* 2022, Merrigan *et al* 2023). However, wearable PPG technology is continually improving due to sensor technology advancements, with each new device iteration potentially offering enhanced accuracy (Kim and Baek 2023, Jena *et al* 2025). Validation of a relatively recent and rugged smartwatch suited for hazardous work, such as the Garmin Instinct 2 (the model used in this study, since then succeeded by the Instinct 3), against a Polar H10 chest strap is promising (Pearce *et al* 2023, McArthur *et al* 2024, Navalta *et al* 2024). However, its validity for using the ECTemp™ algorithm during routine construction work remains unexamined.

The first goal of this study was to assess the use of the ECTemp™ algorithm in estimating *T*_PILL_ among construction workers during a single work shift in the summer. This was accomplished by comparing ECTemp™ obtained from the Polar H10 chest strap (ECTemp_POLAR_) compared to *T*_PILL_. We hypothesized that ECTemp_POLAR_ will provide a reasonably accurate measure of *T*_PILL_. The second goal of this study was to assess the comparative validity of the Garmin Instinct 2 smartwatch against the Polar H10. This involved three comparisons: (1) Garmin Instinct 2-derived ECTemp (ECTemp_GARMIN_) against *T*_PILL_, (2) Garmin Instinct 2-derived heart rate (HR_GARMIN_) against Polar H10-derived heart rate (HR_POLAR_), and (3) ECTemp_GARMIN_ against ECTemp_POLAR_. We hypothesized that (1) ECTemp_GARMIN_ will provide similar accuracy to ECTemp_POLAR_ in estimating T_PILL_ and (2) HR_GARMIN_ and ECTemp_GARMIN_ will show agreement with HR_POLAR_ and ECTemp_POLAR_, respectively.

## 2. Methods

### 2.1. Participants and ethical considerations

The present study involves data collected during two field studies conducted at different commercial construction sites in the Midwest U.S. for eight days total (three days in July 2023 and five days in August 2024). The 2023 study was approved by the University of New Mexico’s Institutional Review Board (protocol #2208012352) and the 2024 study was approved by the Institutional Review Board at Indiana University (protocol #23231). Both studies conformed to the Declaration of Helsinki. More information can be found on the 2023 field study in previous publications (Specht *et al* 2025, Tourula *et al* 2025).

A total of 102 workers were recruited on the job sites via word of mouth, flyers and on-site recruitment events. Interested workers were instructed to meet at a dedicated location on the worksite to discuss the study with research staff, be informed of the experimental procedures and possible risks before providing informed written consent. Workers were excluded for: age < 18 years, weight < 40 kg, pregnancy, pacemaker or electrical medical implant, current or history of intestinal disorders that can lead to obstruction of the digestive tract, including gastroparesis, history of diverticula, history of surgical procedures in the gastrointestinal tract, swallowing disorders, strong electromagnetic field during the period of use of the system (MRI in particular), history of Crohn’s disease and/or irritable bowel syndrome. Physical characteristics were recorded during enrollment and can be found in table 1.

**Table 1.** Participant characteristics.

		2024 cohort (*n* = 77)		
Parameter	2023 cohort polar H10 (*n* = 25)	Polar H10 only (*n* = 17)	Garmin Instinct 2 and Polar H10 (*n* = 60)*	All workers (*n* = 102)
Sex	3 females	1 female	3 females	7 females
Age, years	34 ± 11	41 ± 11	39 ± 11	38 ± 11
Height, cm	175 ± 11	171 ± 6	172 ± 8	173 ± 8
Weight, kg	87.2 ± 17.7	86.6 ± 15.6	92.9 ± 17.7	90.4 ± 17.4
Body mass index, kg m^−2^	28.5 ± 5.1	29.8 ± 5.9	31.4 ± 5.4	30.4 ± 5.5

*Note:* Data are presented as mean ± SD. * *n* = 58 for weight and BMI data in the Garmin Instinct 2 and Polar H10 group.

### 2.2. Procedures

**Figure 1.**
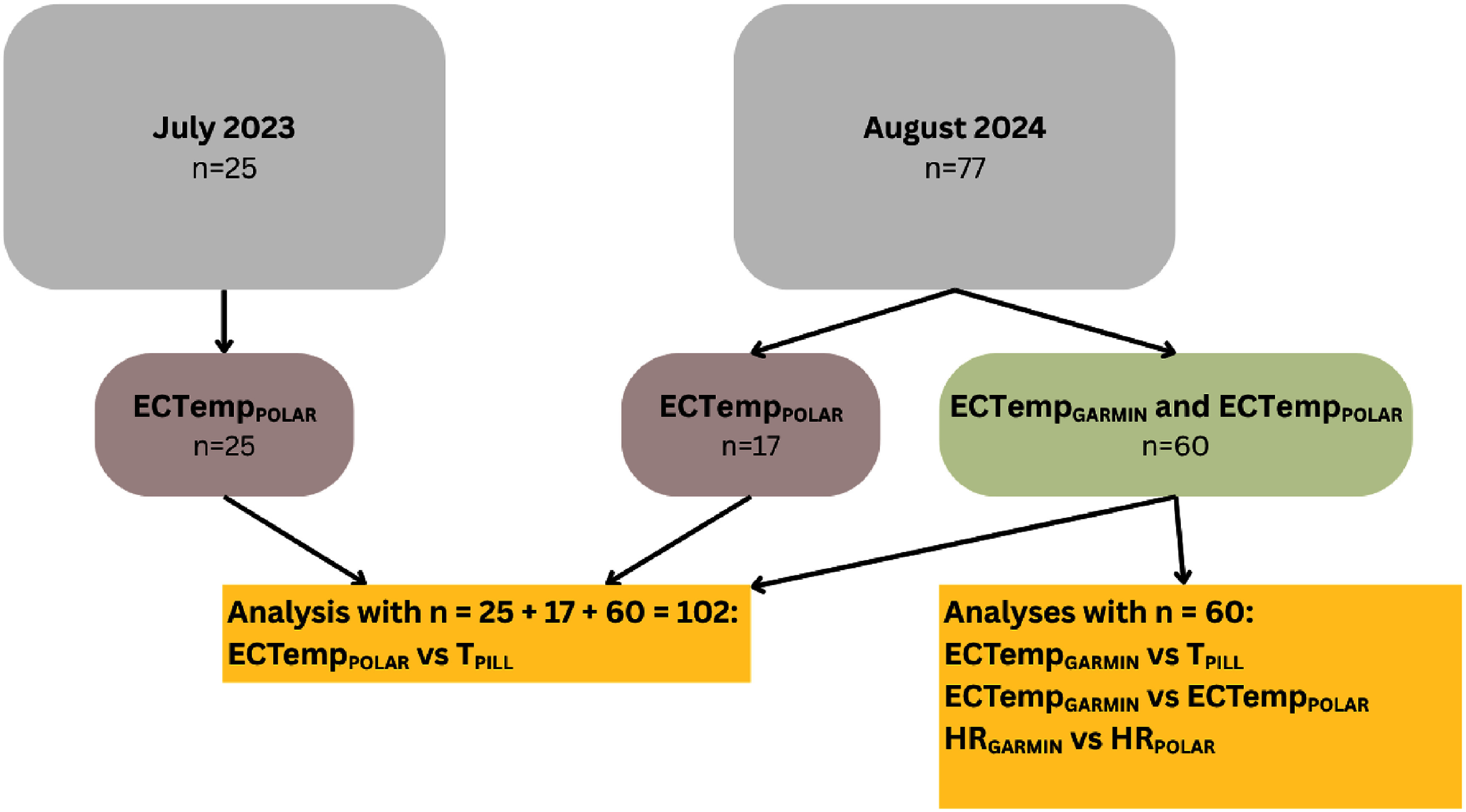
Flow chart representing the participant groups, devices used, and the four comparisons performed. All 102 subjects had measures of *T*_PILL_.

Enrolled workers were instructed to arrive at the dedicated onsite location approximately 30 min before their work shift start time (usually between 3:30 and 5:30 AM). Upon arrival, workers ingested temperature-sensitive pills and were instrumented with a Polar H10 heart rate chest strap monitor and/or a Garmin Instinct 2 smartwatch (table 1 and figure 1). Following instrumentation, workers were encouraged to perform their normal work duties for the duration of their shift. Following completion of their shift, the workers came back for device removal and data downloading.

### 2.3. Environmental conditions

The environmental conditions at each worksite were characterized using data from the nearest standardized personal weather station (www.wunderground.com/). Personal weather stations provide an advantage over other fixed regional weather stations (e.g. those located at an airport) in that they are closer to the jobsites (Wang *et al* 2019, Marquès and Messier 2025). This allowed for consistent reporting across sites and days (table 2).

**Table 2.** Environmental conditions.

Day	Maximal temperature (°C)	Relative humidity at maximal temperature (%)

2023		
1	26.7	76
2	28.9	77
3	30.6	52
Mean ± SD	28.7 ± 2.0	68 ± 14

2024		

1	22.2	84
2	31.7	61
3	31.1	69
4	31.7	57
5	30.6	55
Mean ± SD	29.4 ± 4.1	65 ± 12
Total Mean ± SD	29.2 ± 3.3	66 ± 12

### 2.4. Instrumentation and measurements

*Gastrointestinal pill temperature (T*_PILL_). Ingestible temperature sensitive pills (eCelcius, BodyCAP, Caen, France) were given to workers prior to their work shift. Immediately post-shift, *T*_PILL_ data were downloaded for processing. *T*_PILL_ collected data every 30 s. However, due to the slow upload speed at the end of the workday being excessive for the participant, *T*_PILL_ data were uploaded as an average over every 5 min interval.

*Heart rate.* Heart rate was continuously measured and grouped into 60 s intervals. Before starting their shift, workers from the 2024 study were equipped with two methods of heart rate measurement: (1) a chest strap-based monitor (Polar H10, Polar Electro Inc., Lake Success, NY) used for its accuracy (Laukkanen and Virtanen 1998, Achten and Jeukendrup 2003); and (2) a rugged smartwatch (Instinct 2, Garmin, Olathe, KS). In all instances, workers were properly fitted for the chest strap and smartwatch by trained members of the study team, and the workers were instructed how to properly wear these devices should they become displaced during the workday. Workers from 2023 were equipped only with a chest strap monitor. Data were collected using the Polar Flow app from Polar^TM^ for the 2023 period and the SafeGuard Live app from VigiLife for the 2024 period. Immediately post shift, data from all devices were downloaded for processing.

### 2.5. Data processing

*Data cleaning*. After downloading the *T*_PILL_ data, any recorded data presenting a zero or *a* ⩾ 0.5 °C fluctuation from the previous interval was excluded via visual inspection of the excel data sheets. Due to the ingestible nature of the temperature-sensitive pills, any food or drinks consumed can influence *T*_PILL_ recordings when the pill is in the stomach (Wilkinson *et al* 2008). As expected, due to the pill’s ingestion 30 min before the shift, a large portion of the erroneous data occurred at the beginning of the shift, likely before the pill had exited the stomach.

*Estimated core temperature (ECTemp^TM^).* ECTemp was calculated using the 2022 version of the ECTemp^TM^ algorithm (Buller *et al* 2013, 2020, Looney *et al* 2018). This algorithm uses a Kalman filter that continuously refines core temperature estimates by blending (1) predictions based on past temperature trends with (2) current heart rate measurements, dynamically adjusting the weight given to each source based on their changing reliability over time (Buller *et al* 2013). A later fitting of a sigmoid curve allowed for more precise estimation on the extremes (Looney *et al* 2018). The 60 s intervals collected from the Garmin Instinct 2 and the Polar H10 were used to calculate ECTemp for both devices. The resulting minute-by-minute ECTemp was then averaged into 5 min bins to align with *T*_PILL_. The use of two devices allowed for two independent calculations of ECTemp during work shifts. In our case, we used a standard baseline core temperature of 37.1 °C with an initial variance of 0.01 °C to account for normal physiological variability as described in Buller *et al* (2013).

ECTemp_POLAR_ vs *T*_PILL_. We studied 25 subjects from 2023 and 77 subjects from 2024. After cleaning the data, we obtained 9878 pairwise complete observations.

ECTemp_GARMIN_ vs *T*_PILL_. Only data from 2024 is included. Sixty subjects were included. In total, we have 5944 pairwise complete observations

HR_GARMIN_ vs *HR*_POLAR_. Only data from 2024 is included. Sixty subjects had complete observations for heart rate data, and we found 6767 pairwise complete observations.

ECTemp_GARMIN_ vs ECTemp_POLAR_. Only data from 2024 is included. Sixty subjects were included. In total, we have 5715 pairwise complete observations.

### 2.6. Statistical analysis

**Figure 2.**
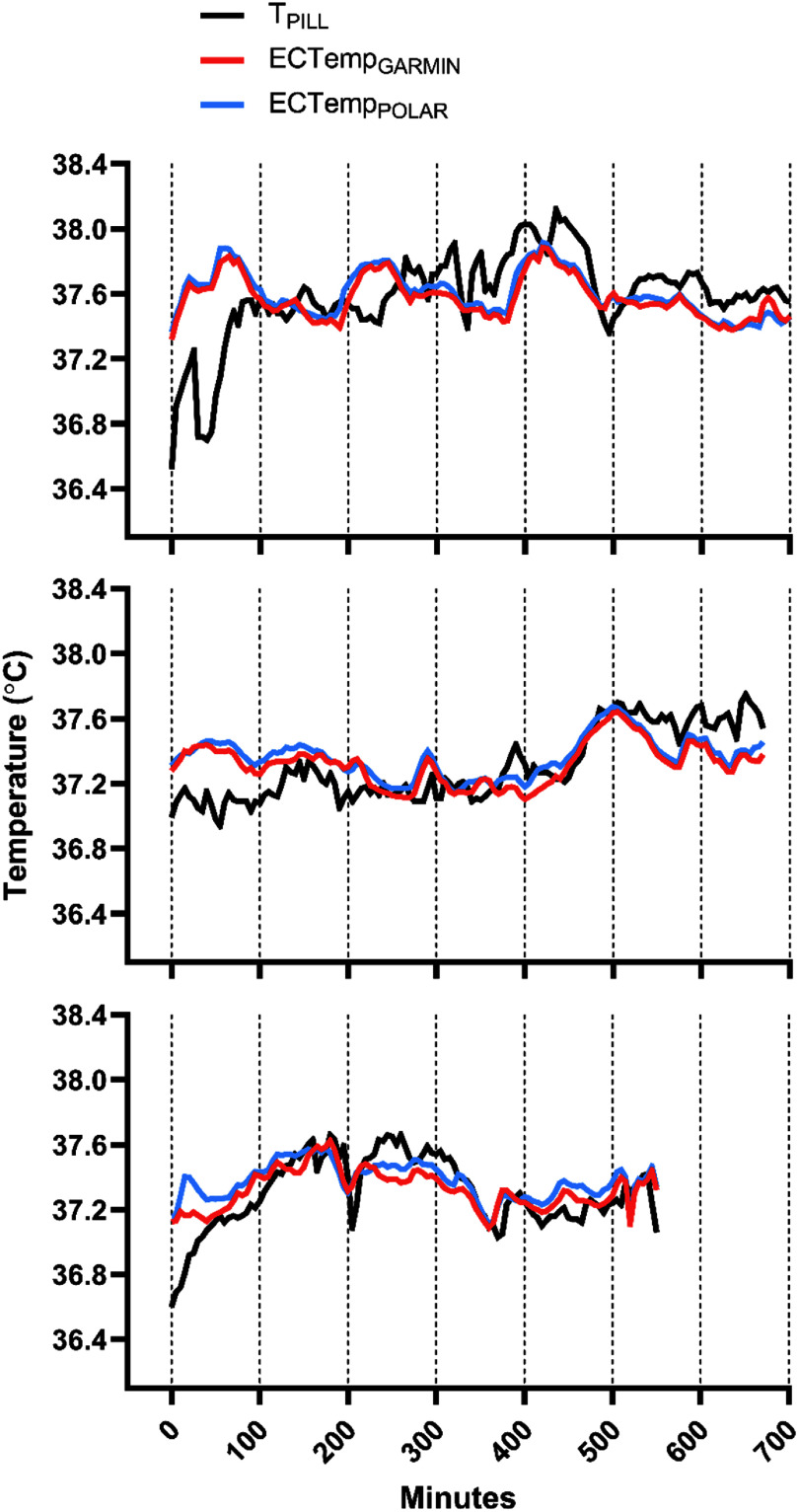
Representative time-series plots of ECTemp_POLAR_, ECTemp_GARMIN_ and *T*_PILL_ for three different workers selected at random over their work shift.

Four different independent analyses were performed to assess the validity of ECTemp in the construction setting and the comparative agreement between measurement devices. First, ECTemp_POLAR_ was compared against *T*_PILL_; second, ECTemp_GARMIN_ was compared against *T*_PILL_; third, HR_GARMIN_ was compared against HR_POLAR_; and lastly, ECTemp_GARMIN_ was compared against ECTemp_POLAR_. A time-series plot of randomly selected workers is presented in figure 2.

For each analysis, the same sequence of statistical tests was applied to the peak values (i.e. the highest *T*_PILL_, ECTemp_POLAR_ or ECTemp_GARMIN_, etc value recorded), mean values, and continuous time-series data. Assessment of agreement between methods was determined using Bland Altman analyses. Mean bias was calculated as the mean of the differences and the 95% limits of agreement were calculated using mean bias ± 1.96 standard deviations of the differences (Bland and Altman 2007). To quantify the strength of the linear relationship between two methods, Pearson correlation coefficients were computed for peak and mean data. Before Pearson correlations were performed, visual inspection of *Q*–*Q* plots was conducted to confirm normality. All peak and mean data sets were normally distributed. Additionally, scatter plots for peak and mean values were visually inspected to confirm linearity and to identify any extreme outliers. No extreme outliers were detected/excluded. Kendall’s Tau correlation coefficients were applied to continuous data, as this non-parametric method does not assume normally distributed data or independence of measurements. Paired t-tests were used to assess the presence of systematic mean differences in the mean and peak values. Lastly, root squared mean error (RMSE) was calculated for the continuous time-series data as a measure of the overall difference between methods.

Analysis of temporal agreement of peak values between devices was determined by identifying the 5 min bin with the highest value. For ease of interpretation, the time difference in minutes between peaks was calculated by counting the number of bins between the peak bins and multiplying by five. Means, standard deviations and 95% confidence intervals of means were used to assess this temporal agreement.

All statistical analyses for physical characteristics, peak, and mean data were conducted using GraphPad Prism version 10.05. The analyses of continuous time-series data were performed using R software (version 4.3.1, R Foundation for Statistical computing).

## 3. Results

**Figure 3.**
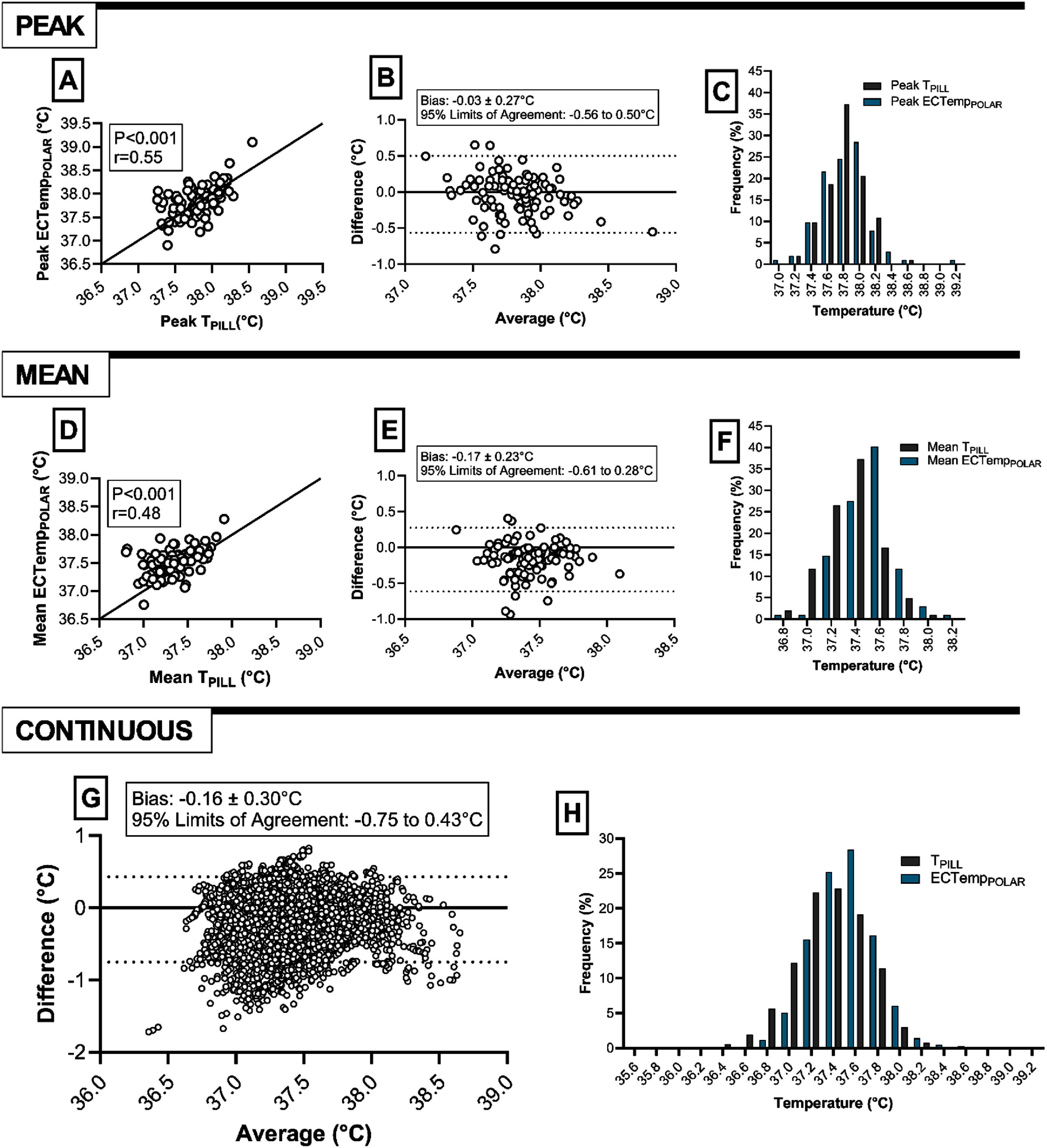
Agreement between ECTemp_POLAR_ and *T*_PILL_. Scatter plots (A), (D), Bland–Altman plots (B), (E), (G), and frequency distribution plots (C), (F), (H) for the ECTemp_POLAR_ vs *T*_PILL_ analysis. Peak values are represented in the first row (A)–(C), mean values in the second row (D)–(F), and continuous time-series values in the third row (G), (H). Scatter plots include a line of identity; Bland–Altman plots show the mean bias (solid line) and 95% limits of agreement (dashed lines). Frequency distributions display the combined frequency of core temperature values measured/estimated by both methods.

*Agreement between ECTemp_POLAR_ vs T_PILL_.* For peak values, the mean bias was −0.03 ± 0.27 °C with 95% limits of agreement from −0.56 °C to 0.50 °C (figure 3(B)). A positive Pearson correlation was observed (*r* = 0.55, *p* < 0.001) (figure 3(A)), and no systematic difference was found via paired *t*-test (mean difference ± SD: 0.03 ± 0.27 °C; *p* = 0.25). For mean values, the mean bias was −0.17 ± 0.23 °C with 95% limits of agreement from −0.61 °C to 0.28 °C (figure 3(E)). A positive Pearson correlation was observed (*r* = 0.48, *p* < 0.001) (figure 3(D)), and a systematic difference was detected via paired *t*-test (mean difference ± SD: 0.17 ± 0.23 °C; *p* < 0.001). For continuous data, the mean bias was −0.16 ± 0.30 °C with 95% limits of agreement from −0.75 °C to 0.43 °C (figure 3(G)). The Kendall’s Tau correlation was *τ* = 0.39 (IQR: 0.23–0.53), with 88.2% of participants showing significant correlations (table 3). The RMSE was 0.32 ± 0.16 °C (figure 7(A)).

**Table 3.** Summary of Kendall’s Tau correlation coefficients for continuous time-series data.

Statistic	ECTemp_POLAR_ vs T_PILL_	ECTemp_GARMIN_ vs T_PILL_	ECTemp_GARMIN_ vs ECTemp_POLAR_	HR_GARMIN_ vs HR_POLAR_
n	102	60	60	60
Median τ	0.39	0.36	0.83	0.75
IQR	[0.23–0.53]	[0.21–0.55]	[0.73–0.90]	[0.66–0.82]
Range	1.1	1.1	0.83	0.52
% Significant (*p* < 0.05)	88.2	90	96.7	100
% *τ* > 0.5	29.4	28.3	95	96.7
% Negative (*τ* < 0)	2.9	3.3	0	0

*Note:* Data are presented as summary statistics of participant-level Kendall’s Tau (*τ*) correlation coefficients. Correlations were calculated individually for each participant’s continuous time-series data. IQR = interquartile range. % Significant indicates the percentage of participants with *p* < 0.05.% *τ* > 0.5 indicates the percentage of participants with ‘strong’ correlations. % Negative (*τ* < 0) indicates the percentage of participants with negative *τ* values (inverse relationship).

**Figure 4.**
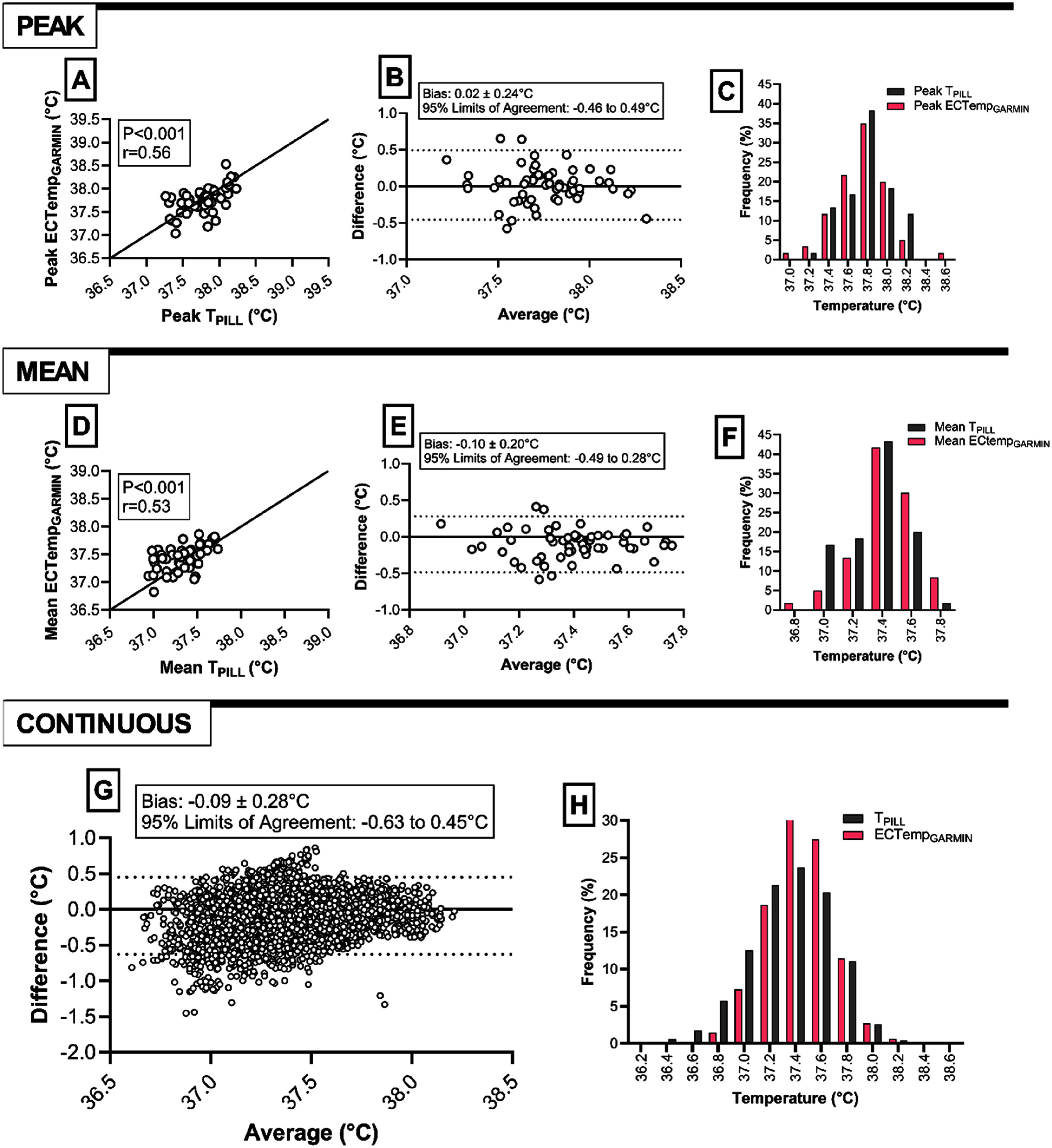
Agreement between ECTemp_GARMIN_ and T_PILL_. Scatter plots (A), (D), Bland–Altman plots (B), (E), (G), and frequency distribution plots (C), (F), (H) for the ECTemp_GARMIN_ vs T_PILL_ analysis. Peak values are represented in the first row (A)–(C), mean values in the second row (D), E, F), and continuous time-series values in the third row (G), (H). Scatter plots include a line of identity; Bland–Altman plots show the mean bias (solid line) and 95% limits of agreement (dashed lines). Frequency distributions display the combined frequency of core temperature values measured/estimated by both methods.

*Agreement between ECTemp_GARMIN_ and T_PILL_.* For peak values, the mean bias was 0.02 ± 0.24 °C with 95% limits of agreement from −0.46 °C to 0.49 °C (figure 4(B)). A positive Pearson correlation was observed (*r* = 0.56, *p* < 0.001) (figure 4(A)), and no systematic difference was found via paired *t*-test (mean difference ± SD: −0.02 ±0.24 °C; *p* = 0.55). For mean values, the bias was −0.10 ± 0.2 °C with 95% limits of agreement from −0.49 °C to 0.28 °C (figure 4(E)). A positive Pearson correlation was observed (*r* = 0.53, *p* < 0.001) (figure 4(D)), and a systematic difference was detected via paired *t*-test (mean difference ± SD: 0.10 ± 0.20 °C; *p* = 0.0001). For continuous data, the mean bias was −0.09 ± 0.28 °C with 95% limits of agreement from −0.63 °C to 0.45 °C (figure 4(G)). The Kendall’s Tau correlation was *τ* = 0.36 (IQR: 0.21–0.55), with 90.0% of participants showing significant correlations (table 3). The RMSE was 0.28 ± 0.12 °C (figure 7(B)).

**Figure 5.**
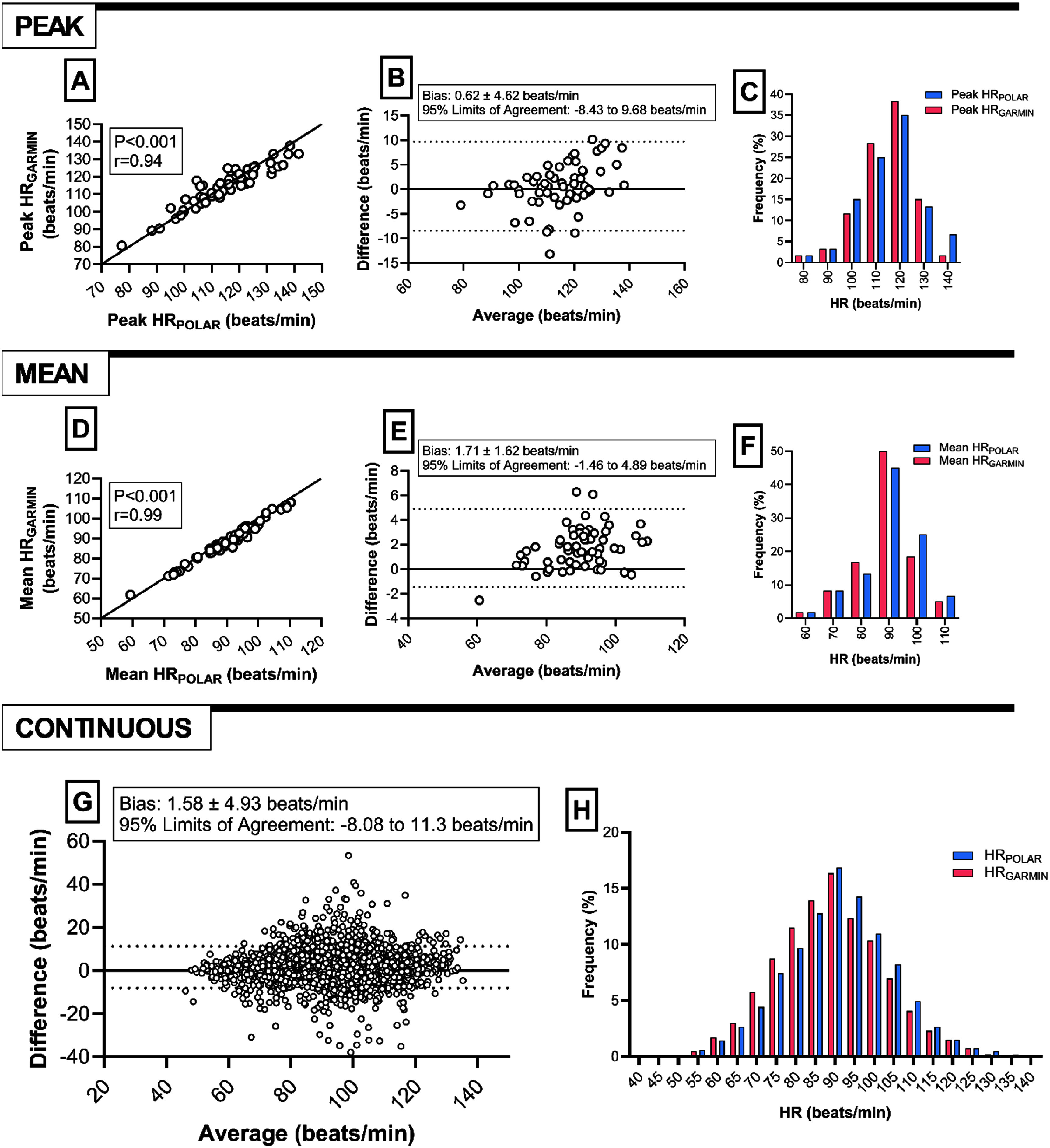
Agreement between HR_GARMIN_ and HR_POLAR_. Scatter plots (A), (D), Bland–Altman plots (B), (E), (G), and frequency distribution plots (C), (F), (H) for the HR_GARMIN_ and HR_POLAR_ analysis. Peak values are represented in the first row (A)–(C), mean values in the second row (D)–(F), and continuous time-series values in the third row (G), (H). Scatter plots include a line of identity; Bland–Altman plots show the mean bias (solid line) and 95% limits of agreement (dashed lines). Frequency distributions display the combined frequency of heart rate values estimated by both methods.

*Agreement between HR_GARMIN_ and HR_POLAR_.* For peak values, the mean bias was 0.6 ± 4.6 beats min^−1^ with 95% limits of agreement from −8.4 beats min^−1^ to 9.7 beats min^−1^ (figure 5(B)). A positive Pearson correlation was observed (*r* = 0.94, *p* < 0.001) (figure 5(A)), and no systematic difference was found via paired *t*-test (mean difference ± SD: −0.6 ± 4.6 beats min^−1^; *p* = 0.30). For mean values, the mean bias was 1.7 ± 1.6 beats min^−1^ with 95% limits of agreement from −1.5 beats min^−1^ to 4.9 beats min^−1^ (figure 5(E)). A positive Pearson correlation was observed (*r* = 0.99, *p* < 0.001) (figure 5(D)), and a systematic difference was detected via paired *t*-test (mean difference ± SD: −1.7 ± 1.6 beats min^−1^; *p* < 0.001). For continuous data, the mean bias was 1.6 ± 4.9 beats min^−1^ with 95% limits of agreement from −8.1 beats min^−1^ to 11.3 beats min^−1^ (figure 5(G)). The Kendall’s Tau correlation was *τ* = 0.75 (IQR: 0.66–0.82), with 100% of participants showing significant correlations (table 3). The RMSE was 4.8 ± 2.1 beats min^−1^ (figure 7(C)).

**Figure 6.**
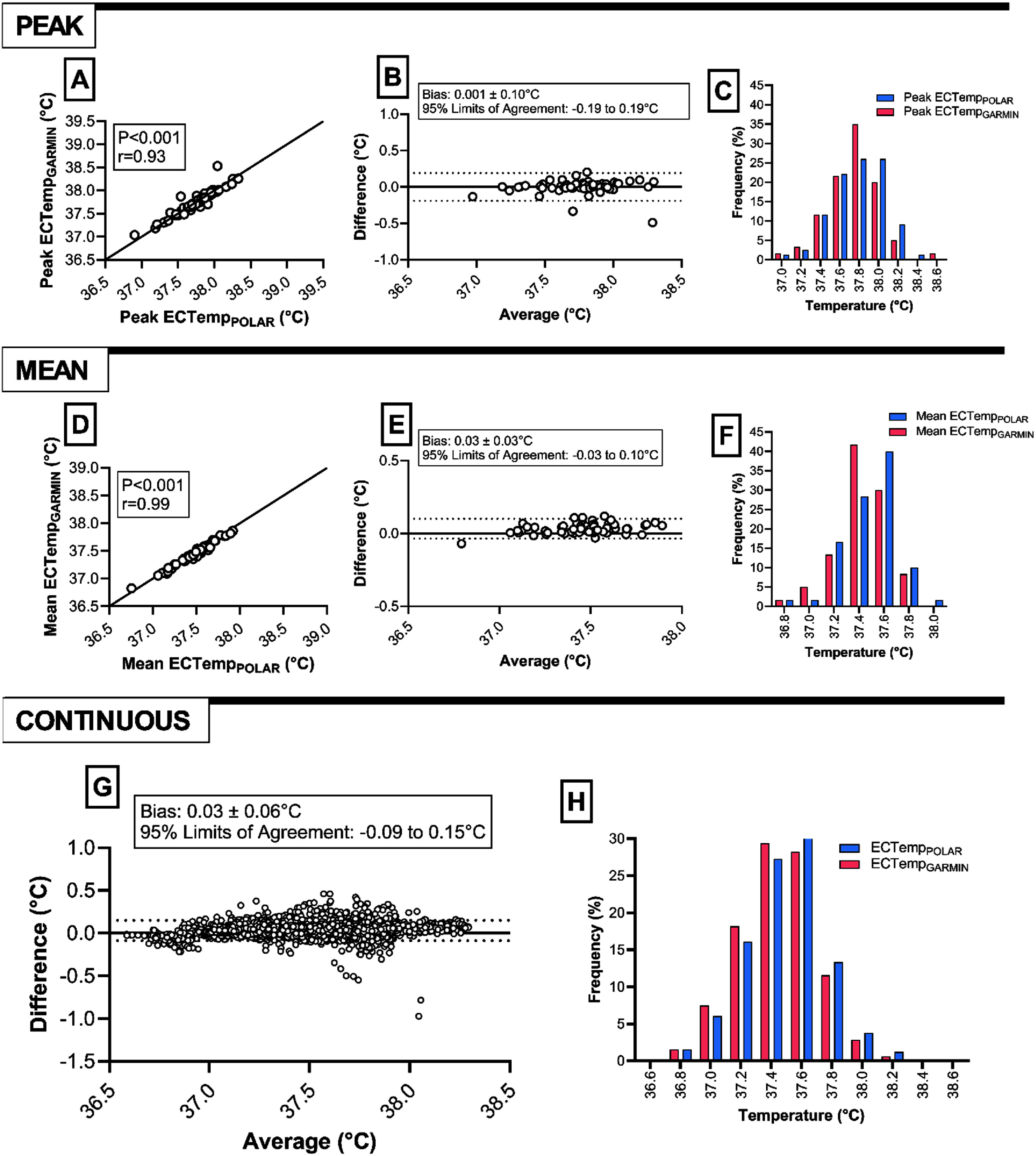
Agreement between ECTemp_GARMIN_ and ECTemp_POLAR_. Scatter plots (A), (D), Bland–Altman plots (B), (E), (G), and Frequency distribution plots (C), (F), (H) for the ECTemp_GARMIN_ and ECTemp_POLAR_ analysis. Peak values are represented in the first row (A)–(C), mean values in the second row (D)–(F), and continuous time-series values in the third row (G), (H). Scatter plots include a line of identity; Bland–Altman plots show the mean bias (solid line) and 95% limits of agreement (dashed lines). Frequency distributions display the combined frequency of core temperature values estimated by both methods.

**Figure 7.**
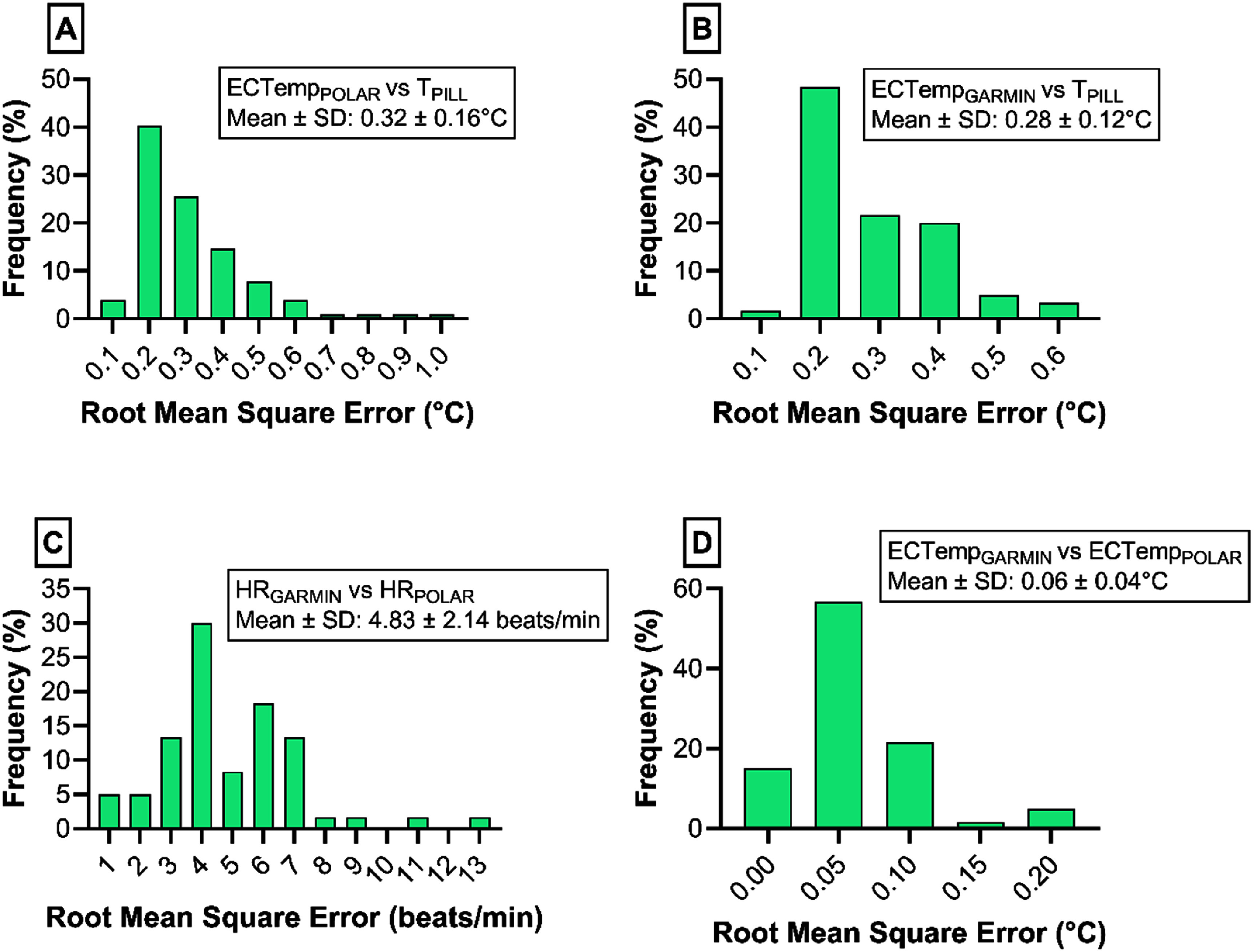
Root mean square error (RMSE) distributions. Histograms of RMSE values for: (A) ECTemp_POLAR_ vs T_PILL_, (B) ECTemp_GARMIN_ vs T_PILL_, (C) HR_GARMIN_ vs HR_POLAR_, and (D) ECTemp_GARMIN_ vs ECTemp_POLAR_, comparisons.

*Agreement between ECTemp_GARMIN_ and ECTemp_POLAR_.* For peak values, the mean bias was 0.001 ± 0.01 °C with 95% limits of agreement from −0.19 °C to 0.19 °C (figure 6(B)). A positive Pearson correlation was observed (*r* = 0.93, *p* < 0.001) (figure 6(A)), and no systematic difference was found via paired *t*-test (mean difference ± SD: −0.001 ± 0.10 °C; *p* = 0.93). For mean values, the mean bias was 0.03 ± 0.03 °C with 95% limits of agreement from −0.03 °C to 0.10 °C (figure 6(E)). A positive Pearson correlation was observed (*r* = 0.99, *p* < 0.001) (figure 6(D)), and a systematic difference was found via paired *t*-test (mean difference ± SD: −0.03 ± 0.03 °C; *p* < 0.001). For continuous data, the mean bias was 0.03 ± 0.06 °C with 95% limits of agreement from −0.09 °C to 0.15 °C (figure 6(G)). The Kendall’s Tau correlation was *τ* = 0.83 (IQR: 0.73–0.90), with 96.7% of participants showing significant correlations (table 3). The RMSE was 0.06 ± 0.04 °C (figure 7(D)).

A comprehensive summary of all agreement metrics for peak, mean, and continuous data is presented in table 4.

**Table 4.** Summary of comparative analysis for peak, mean and continuous time-series data.

Peak data	ECTemp_POLAR_ vs T_PILL_	ECTemp_GARMIN_ vs T_PILL_	ECTemp_GARMIN_ vs ECTemp_POLAR_	HR_GARMIN_ vs HR_POLAR_
Mean bias (±SD)	−0.03 ± 0.27 °C	0.02 ± 0.24 °C	0.001 ± 0.01 °C	0.6 ± 4.6 beats min^−1^
95% limits of agreement	−0.56–0.50 °C	−0.46–0.49 °C	−0.19–0.19 °C	−8.4–9.7 beats min^−1^
Pearson correlation (r)	0.55	0.56	0.93	0.94
Paired *t*-test (p)	0.25	0.55	0.93	0.30

Mean data				

Mean bias (±SD)	−0.17 ± 0.23 °C	−0.10 ± 0.20 °C	0.03 ± 0.03 °C	1.7 ± 1.6 beats min^−1^
95% limits of agreement	−0.61–0.28 °C	−0.49–0.28 °C	−0.03–0.10 °C	−1.5–4.9 beats min^−1^
Pearson correlation (r)	0.48	0.53	0.99	0.99
Paired *t*-test (p)	<0.001	0.0001	<0.001	<0.001

Continuous time-series datap				

Mean bias (±SD)	−0.16 ± 0.30 °C	−0.09 ± 0.28 °C	0.03 ± 0.06 °C	1.6 ± 4.9 beats min^−1^
95% limits of agreement	−0.75–0.43 °C	−0.63–0.45 °C	−0.09–0.15 °C	−8.1–11.3 beats min^−1^
Kendall’s Tau (*τ*) [IQR]	0.39 [0.23–0.53]	0.36 [0.21–0.55]	0.83 [0.73–0.90]	0.75 [0.66–0.82]
% significant correlations	88.20%	90.00%	96.70%	100%
RMSE (±SD)	0.32 ± 0.16 °C	0.28 ± 0.12 °C	0.06 ± 0.04 °C	4.8 ± 2.1 beats min^−1^

*Note:* All Pearson correlations (*r*) were significant at *p* < 0.001.

The temporal alignment of peak values across devices is shown in table 5.

**Table 5.** Mean difference in peak timing between devices.

Statistic	ECTemp_POLAR_ vs *T*_PILL_	ECTemp_GARMIN_ vs *T*_PILL_	ECTemp_GARMIN_ vs ECTemp_POLAR_
N	102	60	60
Mean ± SD	13.8 ± 159 min	−3.67 ± 158 min	5.5 ± 136 min
95% CI of mean	−17.4–45 min	−44.4–37.1 min	−29.6–40.6 min

*Note:* Peak time for each device was determined by identifying the 5 min bin with the highest value. The time differences in minutes were calculated by counting the number of bins between the peak bins and multiplying by 5. Positive values indicate that the peak of the tested device occurred after the peak of the criterion measure, while negative values indicate the peak occurred before the criterion measure. For the first two rows, *T*_PILL_ served as the criterion measure. For the last row, ECTemp_POLAR_ served as the criterion measure.

## 4. Discussion

This first goal of this study was to determine the accuracy of the ECTemp™ algorithm in construction workers during a single work shift, while the second goal was to assess the use of a smartwatch as a heart rate measuring device to feed into the ECTemp^TM^ algorithm. In support of our hypotheses, both ECTemp_POLAR_ and ECTemp_GARMIN_ provide an accurate measure of T_PILL_ during construction work as assessed by the low bias relative to other validation studies (discussed further below). Of particular interest, for continuous time series data, the bias was lower with ECTemp_GARMIN_. Additionally, comparisons showed negligible bias between HR_GARMIN_ and HR_POLAR_ and therefore between ECTemp_GARMIN_ and ECTemp_POLAR_.

Comparison of ECTemp_POLAR_ continuous time series data against *T*_PILL_ yielded −0.16 ± 0.30 °C bias with limits of agreement from −0.75 °C to 0.43 °C (figure 3(G)). This result is consistent with the direction of bias and limits of agreement seen in other validations of the ECTemp^TM^ algorithm, albeit being more negative than the −0.03 °C value reported in the seminal study of Buller *et al* (2013), while also sitting at the lower end of subsequent studies (Buller *et al* 2015, 2020, Looney *et al* 2018, Hagen *et al* 2020, de Korte *et al* 2022, Egbert *et al* 2022, Peggen *et al* 2024). A non-exhaustive summary of ECTemp™ algorithm validation studies is presented in table 6. This table updates the table found in Buller *et al* (2018), incorporating results from recent validations in different settings. In the present study, ECTemp_POLAR_ seems to slightly underestimate core temperature as measured by *T*_PILL_. Nonetheless, the positive participant-level correlation (Kendall’s *τ* = 0.39) (table 3) suggests that ECTemp_POLAR_ tracks the directional changes in *T*_PILL_ with mild variability. The RMSE of 0.32 ± 0.16 °C (figure 7(A)) is consistent with previous publications and resides below the 0.5 °C threshold for real time thermoregulatory models proposed in other publications (Yokota *et al* 2012, 2014).

**Table 6.** Summary of ECTemp™ validation study results compared to a core temperature reference.

Study	n	Bias (°C)	Limits of agreement (°C)	RMSE (°C)	Population or setting
Buller *et al* (2020)—“WARM group”	11	+0.34	−0.44–1.12	0.49	Military
de Korte *et al* (2022)	101	+0.15	−0.30–0.60	0.35	Elite athletes

Peggen *et al* (2024)	18	+0.09	−0.35–0.53	0.37	Prolonged walking
Buller *et al* (2015)	27	+0.02	−0.46–0.50	0.21	Military (PPE)
Hunt *et al* (2019)	8	+0.01	−0.63–0.65	0.32	Explosive ordnance disposal PPE

Showers *et al* (2016)	33	−0.01	−0.59–0.57	0.29	Military
Buller (2015)	16	−0.01	−0.63–0.61	0.28	Military
Buller *et al* (2013)	89	−0.03	−0.66–0.60	0.30	Various (model validation)
Buller *et al* (2016)	95	−0.07	−0.58–0.44	0.22	
**ECTemp_GARMIN_**	**60**	**−0.09**	**−0.63–0.45**	**0.28**	**Construction workers**

Buller *et al* (2020)—“HOT group”	10	−0.10	−0.83–0.63	0.37	Military
Hagen *et al* (2020)	13	−0.11	−1.00–0.79		American football athletes
Looney *et al* (2018)—Validation group, sigmoid	8	−0.13	−0.64–0.38	0.29	Military
Egbert *et al* (2022)	35	−0.14	−0.90–0.62	0.41	Agricultural workers
Buller *et al* (2016)	43	−0.15	−0.52–0.22	0.22	
**ECTemp_POLAR_**	**102**	**−0.16**	**−0.75–0.43**	**0.32**	**Construction workers**

*Note:* Studies are ordered by mean bias (most positive to most negative) and studies included are non-exhaustive. Limits of agreement represent the 95% range (mean bias ± 1.96 × SD of differences). RMSE = root mean square error. Data for Hagen *et al* (2020) were converted from Fahrenheit to Celsius. The results from the present study are included in bold for context. Single black line equals a hypothetical bias of 0 and delimits positive bias from negative bias. Smaller double black lines delimit studies that are within the proposed clinical threshold of <0.1 (Gant *et al* 2006, Byrne and Lim 2007) (this threshold is indicative only and does not signify that the device is not applicable for in-the-fields settings). This table extends a rearranged table 1 presented in Buller *et al* (2018).

When compared to *T*_PILL_, ECTemp_GARMIN_ continuous time series data showed a bias of −0.09 ± 0.28 °C (figure 4(G)), which is consistent with prior research that utilized chest strap based monitors (table 6) (Buller *et al* 2013, 2015, Hunt *et al* 2019, Hagen *et al* 2020, Egbert *et al* 2022, Peggen *et al* 2024) and is comparable to the bias we observed for ECTemp_POLAR_ presented in figure 3(G). This degree of bias is similar to the one seen when comparing *T*_PILL_ against clinical core temperature gold standards methods (rectal or esophageal temperature) (Patel *et al* 1996, Lee *et al* 2000, Lefrant *et al* 2003, Casa *et al* 2007). As such, since *T*_PILL_ seems to have an inherent bias (Casa *et al* 2007, Travers *et al* 2016), interpretation of the comparatively low ECTemp_GARMIN_ bias seen in our study should acknowledge *T*_PILL_’s characteristic error. Nonetheless, the mean bias for ECTemp_GARMIN_ continuous time series data falls below the threshold mean bias of 0.1 °C deemed acceptable for core temperature measures in a clinical setting (Gant *et al* 2006, Byrne and Lim 2007, Peggen *et al* 2024). While a clinical application is outside the scope of field monitoring in an occupational scenario, this level of agreement indicates that ECTemp_GARMIN_ may meet sufficient validity in a construction work setting, potentially allowing for future longitudinal assessment using ECTemp^TM^. However, the 95% limits of agreement (−0.63 °C to 0.45 °C) and RMSE (0.28 ± 0.12 °C) seen for ECTemp_GARMIN_ and ECTemp_POLAR_ (95% limits of agreement: −0.75 °C to 0.43 °C; RMSE: 0.32 ± 0.16 °C) indicate substantial individual-level variability in measurement accuracy. In both devices, the reported individual- level variability is consistent with those reported in validation studies across various settings (table 6) (Buller *et al* 2013, 2015, 2020, Looney *et al* 2018, Hagen *et al* 2020, de Korte *et al* 2022, Egbert *et al* 2022, Peggen *et al* 2024). This suggests that ECTemp may be best used at a group level to track core temperature trends over a work shift, which could be used to promote timely intervention, such as rest-shade-hydration or the use of other cooling modalities. Future users of ECTemp^TM^ in construction workers should therefore consider the apparent wide individual-level variability in their field-based decision-making.

The presence of two types of heart rate monitors provided a direct comparison between the PPG-based smartwatch and the validated chest strap (Laukkanen and Virtanen 1998, Achten and Jeukendrup 2003) in a construction work setting (figures 5 and 6). Continuous time series data between HR_GARMIN_ and HR_POLAR_ yielded a bias of 1.6 ± 4.9 beats min^−1^ with limits of agreement of −8.1 beats min^−1^ to 11.3 beats min^−1^ (figure 5(G)) which stand in contrast to the larger bias (5.3 beats min^−1^) and considerably wider limits of agreement (−17.1–27.8 beats min^−1^) reported by Navalta *et al* (2024) for the same devices during simulated pickleball play. This discrepancy may be explained by the fundamentally different dynamic activities involved. It has been suggested that activities like pickleball introduce a lot of movement artifacts in PPG devices due to arm swings (Hermand *et al* 2019, Alfonso *et al* 2022, López‐Belmonte *et al* 2023, Navalta *et al* 2024, Schweizer and Gilgen-Ammann 2025). To that effect, it is plausible that certain construction tasks with high upper-body movement (e.g. hammering, drilling, overhead work, etc) could also introduce movement artifacts, though likely of a different nature than pickleball. Moreover, there is speculation that the Garmin Instinct 2 may not be valid in measuring heart rate data closer to maximal heart rate (Pearce *et al* 2022, Zarei *et al* 2023, Navalta *et al* 2024). This seems to not be a concern in the context of our study, as the peak heart rate bias was even lower (0.6 ± 4.6 beats min^−1^; figure 5(B)) than the mean bias (1.7 ± 1.6 beats min^−1^; figure 5(E)) and continuous bias (1.6 ± 4.9 beats min^−1^; figure 5(G)), but it is unlikely that our values came close to maximal heart rate. Thus, the apparent lower than maximal peak heart rate encountered during the workdays of observation may have artificially increased the accuracy of this device. For context, the peak mean heart rate of 114.8 beats min^−1^ while using the Garmin Instinct 2 in the present study is smaller than the average mean of 124.4 beats min^−1^ during pickleball reported in Navalta *et al* (2024) and the peak of 152 beats min^−1^ during pickleball using another device in Denning *et al* (2022). When considered on a broader scale of wearable devices, the Garmin Instinct 2 biases in this study are on the lower end relative to the biases observed in various settings and exercise intensities as reported in other studies (Pasadyn *et al* 2019, Støve *et al* 2019, Navalta *et al* 2020, 2023, 2024, Muggeridge *et al* 2021, Merrigan *et al* 2023).

Finally, the level of bias between ECTemp_GARMIN_ and ECTemp_POLAR_ (bias: 0.03 ± 0.06 °C; limits of agreement: −0.09 °C to 0.15 °C; figure 6(G)) demonstrates that the smartwatch performs well in generating ECTemp^TM^-derived core temperature values. This is consistent with the bias seen between devices for heart rate data (figure 5(G)) as ECTemp^TM^ is derived from heart rate.

## 5. Considerations

Some limitations should be considered when interpreting the data presented. First, the sample size for analyses involving the Garmin Instinct 2 (*n* = 60) was smaller than that of the Polar H10 (*n* = 102), which may influence biases and limits of agreement. Second, while *T*_PILL_ is a valid and widely used surrogate for core temperature (Bongers *et al* 2018), it can be influenced by the ingestion of food and cold fluids (Wilkinson *et al* 2008) and has a known inherent bias when compared to rectal temperature (Travers *et al* 2016). Thirdly, an assessment of the validity of ECtemp^TM^ at greater levels of hyperthermia could not be made due to the relatively low peak *T*_PILL_ (across all workers; peak *T*_PILL_ did not exceed 39.1 °C, with 90% of peak values below 38.2 °C). Fourthly, poor or loose fit and some tattoos may limit the accuracy of the smart watch measurement of heart rate. The researchers addressed and mitigated these limitations by instrumenting each worker to ensure a proper fit and avoid any tattoo skin and the workers were instructed how to properly wear these devices should they become displaced during the workday. That said, proper fit could not be formally assessed throughout the workday. Fifthly, while the female-to-male ratio in our dataset is representative of what is typically seen in the construction sector (Labor Force Statistics from the Current Population Survey 2026), females are heavily underrepresented within our analysis. Sixthly, because *T*_PILL_ data was collected in 5 min bins, ECTemp was calculated at 1 min resolution then averaged to 5 min bins for alignment. This temporal aggregation could potentially affect the dynamic responsiveness of the algorithm and influence agreement metrics. This is perhaps highlighted by the large variability in the timing of the peak *T*_PILL_/ECTemp observations (table 5) and modest Kendall *τ* values (table 3). Although no significant differences were observed for peak values using paired t-tests, mean values showed a significant difference (table 4). We speculate that this difference occurred because mean-based outcomes integrate information across many time points within each participant, which can reduce variability and thereby increase statistical power relative to peak-based outcomes that rely on a single observation per participant. Consequently, the t-test results should be interpreted alongside other reported agreement metrics, particularly given the potential for temporal misalignment of peak values. Finally, while the field-based nature of this study introduced uncontrolled factors (individual work tasks, hydration status, and self-paced work rates) that may induce variation in the physiological state between workers, the within subject nature of our analysis allows for a direct comparison of devices.

## 6. Perspectives and significance

This study indicates that the ECTemp™ algorithm provides an adequate estimation of core temperature for use in construction settings. For a practical field application, the Garmin Instinct 2 smartwatch could be used as an alternative to chest straps, as the level of agreement with *T*_PILL_ observed is consistent with other field validations (table 6) and falls below the 0.1 °C threshold. Given the aforementioned individual-level variability indicated by the limits of agreement, these devices should be used at the group level rather than at an individual level. This could be used alongside monitoring of other important factors dictating WrHS, e.g. Wet Bulb Globe Temperature (Budd 2008).

Recent work has proposed enhanced Kalman filter-based models for core temperature estimation using heart rate. For example, Zhao and Bergmann (2025) combined a Kalman filter with a long short-term memory network which corrects observation residuals, while Han *et al* (2025) added a long-sequence forecasting deep learning model that showed further error reduction when skin temperature data was incorporated. These examples suggest that core temperature estimations could be further refined for field monitoring, possibly by incorporating additional physiological measures. Further work is required.

## 7. Conclusions

This study provides a novel validation of the ECTemp™ algorithm in construction workers and a direct comparison of two wearable devices for this application. Our findings demonstrate that the algorithm provides a reasonable estimate of group-level core temperature, with ECTemp_GARMIN_ potentially providing an equally valid but more practical source of core temperature estimation. Future research should consider incorporating the use of modern, rugged, and validated PPG-based smartwatches as a tool for longitudinal assessment in the construction workforce.

## Data Availability

The data cannot be made publicly available upon publication because they contain sensitive personal information. The data that support the findings of this study are available upon reasonable request from the authors.
